# Renal tubular acidosis is highly prevalent in critically ill patients

**DOI:** 10.1186/s13054-015-0890-0

**Published:** 2015-04-06

**Authors:** Richard Brunner, Andreas Drolz, Thomas-Matthias Scherzer, Katharina Staufer, Valentin Fuhrmann, Christian Zauner, Ulrike Holzinger, Bruno Schneeweiß

**Affiliations:** Department of Medicine III – Division of Gastroenterology and Hepatology, Medical University of Vienna, Waehringer Guertel 18-20, Vienna, 1090 Austria

## Abstract

**Introduction:**

Hyperchloremic acidosis is frequent in critically ill patients. Renal tubular acidosis (RTA) may contribute to acidemia in the state of hyperchloremic acidosis, but the prevalence of RTA has never been studied in critically ill patients. Therefore, we aimed to investigate the prevalence, type, and possible risk factors of RTA in critically ill patients using a physical-chemical approach.

**Methods:**

This prospective, observational trial was conducted in a medical ICU of a university hospital. One hundred consecutive critically ill patients at the age ≥18, expected to stay in the ICU for ≥24 h, with the clinical necessity for a urinary catheter and the absence of anuria were included.

Base excess (BE) subset calculation based on a physical-chemical approach on the first 7 days after ICU admission was used to compare the effects of free water, chloride, albumin, and unmeasured anions on the standard base excess. Calculation of the urine osmolal gap (UOG) - as an approximate measure of the unmeasured urine cation NH_4_^+^ - served as determinate between renal and extrarenal bicarbonate loss in the state of hyperchloremic acidosis.

**Results:**

During the first week of ICU stay 43 of the patients presented with hyperchloremic acidosis on one or more days represented as pronounced negative BE_Chloride_. In 31 patients hyperchloremic acidosis was associated with RTA characterized by a UOG ≤150 mosmol/kg in combination with preserved renal function. However, in 26 of the 31 patients with RTA metabolic acidosis was neutralized by other acid-base disturbances leading to a normal arterial pH.

**Conclusions:**

RTA is highly prevalent in critically ill patients with hyperchloremic acidosis, whereas it is often neutralized by the simultaneous occurrence of other acid-base disturbances.

**Trial registration:**

Clinicaltrials.gov NCT02392091. Registered 17 March 2015

## Introduction

Imbalances of the acid-base state and particularly metabolic acidosis are common problems in critically ill patients. To further investigate the underlying causes, analysis of the acid-base state and determination of plasma and urine electrolytes are crucial [1]. Metabolic acidosis is subdivided in conditions with either high serum anion gap (SAG) or normal SAG according to the classic model of metabolic acid-base disorders. The SAG is the difference in the measured cations (Na^+^, K^+^) and anions (Cl^−^, HCO^3−^) in serum.

High SAG is typical for the occurrence of unmeasured anions (for example ketones, lactate, metabolites of methanol and ethylene glycol, phosphate) where HCO^3−^ is consumed via its action as a buffer. High SAG metabolic acidosis is seen in ketoacidosis, lactic acidosis, intoxication and acute renal failure [2].

Metabolic acidosis with normal SAG - hyperchloremic acidosis - is characterized by the replacement of bicarbonate with chloride caused by extrarenal or renal bicarbonate disposal or a dysfunction in renal H^+^ secretion [1]. Hyperchloremic acidosis is typically seen in bicarbonate wasting due to diarrhea, urine-gut diversions, intestinal or pancreatic fistulae, surgical drains or renal tubular acidosis (RTA) [3].

The analysis of the urine osmolal gap (UOG) is necessary to further differentiate these conditions. The UOG represents the difference of the directly measured urine osmolality to an estimate of the urine osmolality derived from the urine concentrations of sodium, potassium, urea nitrogen, and glucose concentrations [4].

A UOG - as an approximate measure of unmeasured urine ammonium salts - of less than 150 mosmol/kg in combination with a preserved renal function [5] is characteristic for altered urine acidification typical for RTA [4,6], while a UOG of more than 150 mosmol/kg suggests extrarenal bicarbonate loss.

The UOG, in contrast to the urine anion gap, is not invalidated by increased excretion of unmeasured anions and therefore is the preferred measure to further analyze the causes for hyperchloremic acidosis in critically ill patients [7,8].

Type 1 (distal) RTA is based on defective distal tubular H+ secretion and is characterized by inadequately high urinary pH (>5.3) during hyperchloremic metabolic acidosis. In type II (proximal) RTA the underlying cause is a defect in proximal tubule bicarbonate reabsorption. This results in urinary bicarbonate loss also resulting in inappropriately high urine pH during hyperchloremic acidosis. However, in established type II RTA, after severe bicarbonate depletion has occurred, minimum urinary pH is usually below pH 5.3. Type IV RTA is based on mineralocorticoid deficiency or inadequate renal response to mineralocorticoids. It is characterized by low urinary pH and - contrary to RTA types I/II - hyperkalemia [3,9].

The prevalence of RTA and underlying metabolic changes has been studied in patients after kidney transplantation [9] and in osteoporotic patients [10]. However, studies in critically ill patients are lacking.

Based on the physical-chemical approach, more than one metabolic acid-base disorder at the same time was found to be common in critical illness [11]. Thus, ‘metabolic acidosis’ and ‘metabolic alkalosis’ may be present in the same patient at the same time, potentially leading to normal pH values. The terms ‘acidosis’ and ‘alkalosis’ are defined as separate effects that decrease or increase arterial pH, respectively, whereas ‘acidemia’ and ‘alkalemia’ are defined as net arterial pH <7.35 or >7.45, respectively.

Therefore, the aim of this study was to determine the prevalence of RTA in critically ill patients and to analyze the underlying acid-base state using a physical-chemical approach according to Gilfix *et al.* [12].

## Materials and methods

### Patients and setting

This prospective, observational trial was conducted in an eight-bed medical ICU at the University Hospital of Vienna, Austria between April and October 2011. One hundred consecutive critically ill patients fulfilling the inclusion criteria (age ≥18, the expectancy to stay in the ICU for ≥24 h, and the clinical necessity for a urinary catheter) were enrolled within 36 hours after ICU admission. Patients expected to stay <24 h and anuric patients were excluded from enrollment.

Data of routinely obtained blood from arterial blood lines and urine samples were collected in all patients up to 7 days after study inclusion. Arterial blood gas analysis (pH, sodium, chloride, potassium, standard bicarbonate, standard base excess (SBE), pCO_2_, and lactate) was performed daily at 05:00 a.m. using a blood gas analyzer (ABL 725, Radiometer, Copenhagen, Denmark). At the same time, arterial plasma concentrations of albumin, creatinine, blood urea nitrogen, hemoglobin, phosphate, and magnesium, as well as spot urine concentrations of sodium, potassium, chloride, osmolality, and urine pH were measured at the hospital’s routine laboratory using a fully automated analyzer (Hitachi 917, Roche Diagnostics GmbH, Mannheim, Germany). Spot urine was obtained by clamping the patient’s urine catheter for one hour. Subsequently, a urine sample was obtained for analysis as described above.

For every patient the following data were routinely documented: age, sex, height, weight, body mass index, admission reason and comorbidities, medication, Acute Physiology and Chronic Evaluation II (APACHE II) score, Simplified Acute Physiology Score II (SAPS II) score, Sequential Organ Failure Assessment (SOFA) score and body temperature. Heart rate, blood pressure, and urinary output were routinely documented every 4 hours and creatinine clearance was calculated every 24 hours.

The study was approved by the Ethics Committee of the Medical University of Vienna. According to the Austrian law and the guidelines of the research ethics committee, written informed consent was obtained from patients after they regained consciousness.

### Acid-base analysis and calculation of urine osmolal gap

The acid-base state was analyzed using a physical-chemical approach [12]. The SBE is influenced by alterations of free water, chloride, albumin, and unmeasured anions. Therefore, SBE is composed of four subsets which were calculated as follows [11].

Base excess (BE) caused by the free water effect:

$$ \mathrm{Base}\ {\mathrm{excess}}_{\mathrm{Sodium}} = 0.3 \times \left({{\mathrm{Na}}^{+}}_{\mathrm{measured}} - {{\mathrm{Na}}^{+}}_{\mathrm{normal}}\right) $$

The acid-base state is altered by chloride. This effect can be obtained by first correcting for changes in free water:

$$ {{\mathrm{Cl}}^{-}}_{\mathrm{Na}+\mathrm{corrected}} = {\mathrm{Cl}}^{-} \times \left(\left({{\mathrm{Na}}^{+}}_{\mathrm{normal}}/{{\mathrm{Na}}^{+}}_{\mathrm{measured}}\right)\right) $$

Base excess changes based on changes in chloride:

$$ \mathrm{Base}\ {\mathrm{excess}}_{\mathrm{Chloride}} = {{\mathrm{Cl}}^{-}}_{\mathrm{normal}}\ \hbox{--}\ {{\mathrm{Cl}}^{-}}_{\mathrm{Na}+\mathrm{corrected}} $$

Albumin is a weak non-volatile acid. The base excess effect due to albumin was calculated as:

$$ \mathrm{Base}\ {\mathrm{excess}}_{\mathrm{Albumin}}=\left(0.148\times {\mathrm{pH}}_{\mathrm{arterial}} - 0.818\right) \times \left({\mathrm{Albumin}}_{\mathrm{normal}} - {\mathrm{Albumin}}_{\mathrm{measured}}\right). $$

$$ {{\mathrm{Cl}}^{-}}_{\mathrm{normal}} = 105\ \mathrm{mg}/\mathrm{dL};\ {{\mathrm{Na}}^{+}}_{\mathrm{normal}} = 140\ \mathrm{mg}/\mathrm{dL};\ {\mathrm{Albumin}}_{\mathrm{normal}} = 42\ \mathrm{g}/\mathrm{L} $$

Further changes in base excess are based on alterations of unmeasured anions like lactate or ketone bodies. Their effect on the base excess was quantified as follows:

$$ \mathrm{Base}\ {\mathrm{excess}}_{\mathrm{unmeasured}\ \mathrm{anions}} = \mathrm{Base}\ \mathrm{excess}\ \hbox{--}\ \left(\mathrm{Base}\ {\mathrm{Excess}}_{\mathrm{Sodium}} + \mathrm{Base}\ {\mathrm{Excess}}_{\mathrm{Chloride}} + \mathrm{Base}\ {\mathrm{Excess}}_{\mathrm{Albumin}}\right) $$

Based on these calculations hyperchloremic acidosis was defined as: base excess attributable to changes of plasma chloride (BE_Chloride_) ≤ −5 mmol/L. Hyperchloremic acidosis is characterized by extrarenal or renal bicarbonate disposal or a dysfunction in renal H^+^ secretion. To further investigate the primary cause of hyperchloremic acidosis the UOG was calculated.

The UOG represents the difference of the directly measured urine osmolality to an estimate of the urine osmolality derived from the urine concentrations of sodium, potassium, urea nitrogen, and glucose concentrations using the following formula [4]: calculated urine osmolality (mosmol/kg) = (2 × (Na^+^ + K^+^)) + (urea nitrogen in mg/dL)/2.8 + (glucose in mg/dL)/18.

The UOG is an approximate measure of unmeasured urine ammonium salts, which generally are the only other major urinary solutes that contribute significantly to the urine osmolality.

The major renal response to metabolic acidosis is increased NH4^+^ excretion. Therefore, a UOG of less than 150 mosmol/kg in combination with a preserved renal function (glomerular filtration rate >25 ml/min [5]) represents an inadequate renal response and is characteristic for RTA [4,6], while a UOG of more than 150 mosmol/kg suggests extrarenal bicarbonate loss.

Subtypes of RTA were determined as follows [3,9]:

RTA type I (distal): hyperchloremic acidosis with a minimum urine pH ≥5.3 and low/normal plasma potassium (<5.5 mmol/L), based on reduced H^+^ secretion in the distal tubule.

RTA type II (proximal): hyperchloremic acidosis with a minimum urine pH <5.3 and low/normal plasma potassium (<5.5 mmol/L), based on reduced HCO^3−^ reabsorption in the proximal tubule.

RTA type IV: hyperchloremic acidosis with a minimum urine pH <5.3 and high plasma potassium (≥5.5 mmol/L), based on reduced H^+^ and K^+^ excretion in the distal tubule.

Transient RTA was defined as RTA on a single study day, while persistent RTA was defined as RTA on two or more days.

### Objectives

The primary objective was to evaluate the prevalence of RTA in critically ill patients during the first week after intensive care unit (ICU) admission, defined as hyperchloremic acidosis (BE_Chloride_) ≤ −5 mmol/L), a UOG of less than 150 mosmol/kg, and a preserved renal function. Secondary outcome measures were to evaluate the risk factors for RTA.

### Statistics

Data are presented as mean ± standard deviation, median (25^th^ to 75^th^ percentile) or absolute count and relative frequency. For bivariate comparisons, we tabulated data and used *t* test, Mann-Whitney *U* test, chi-squared or Fisher's exact test as appropriate to test the null hypothesis of no difference.

To assess the predictive value of potential risk factors on the incidence of RTA we performed a binary logistic regression analysis with RTA as outcome and diabetes, hypertension, congestive heart failure, chronic kidney disease, liver cirrhosis, neuroleptic drugs, muscle relaxants, combined antibiotic schemes, plasmapheresis, and sedoanalgesia, as well as age, body mass index (BMI), gender, and severity of illness (SAPS II) as co-factors.

To assess the effect of transient versus persistent RTA we conducted four multivariate regression analyses with the outcomes ‘length of ICU stay’, ‘length of hospital stay’, ‘ICU survival’ or ‘hospital survival’ and the co-factors RTA (persistent vs. transient) and ‘number of study days’.

For data management and statistical analyses we used Microsoft Excel 2010 and IBM SPSS (version 20) for Windows (IBM Corp, Armonk, NY, USA).

## Results

In this prospective, observational trial we included 100 critically ill patients (Table 1), which were evaluated daily for hyperchloremic acidosis and RTA during the first week after ICU admission. In total, 373 complete data sets - each representing one patient day - derived from arterial blood and spot urine samples were analyzed.

**Table 1:** **Admission reason and patients’ characteristics**

**Admission reason**	**N = 100**
Respiratory failure	31
St.p. Cardiopulmonary resuscitation	21
Sepsis/septic shock	8
Cardiogenic/hypovolemic shock	4
Coma	5
Esophageal/GI bleeding	6
Acute liver failure	1
Intoxication	1
Necrotizing pancreatitis	1
Postoperative	21
Diabetes mellitus	25
Arterial hypertension	41
Chronic renal failure	18
Liver cirrhosis	11
Congestive heart failure	45
Acute or acute-on-chronic renal failure	13
Nephrotoxic medication during study period	93
Amphotericin B	4
Neuroleptic drugs	13
Combined antibiotic schemes	37
Plasmapheresis	3
Sedoanalgesia	55
Muscle relaxants	3
Calcineurin inhibitors	5
Shock during study period	57
Sepsis during study period	42
Mechanical ventilation during study period	73
Age (years)	62 ± 16
Gender (female/male)	40/60
BMI (kg/m^2^)	26 ± 5
SOFA score	8 ± 4
APACHE II score	20 ± 8
SAPS II score	51 ± 20
Length of ICU stay (days)	6 (3 - 11)
ICU mortality (non-survivors)	14
Serum creatinine^*^ (mg/dL)	1.42 ± 0.88
Blood urea nitrogen^*^ (mg/dL)	30 ± 22
Serum uric acid^*^ (mg/dL)	5.2 ± 2.5
Serum phosphate^*^ (mg/dL)	1.12 ± 0.37
Arterial pH^*^	7.37 ± 0.09
Standard bicarbonate^*^ (mmol/L)	24.8 ± 4.7
Serum anion gap^*^ (mmol/L)	11 ± 3.7
Urine anion gap^*^ (mmol/L)	43 ± 42
Creatinine clearance^#^ (mL/min)	66 (33 - 108)
Urine volume^#^ (in 24 h)	1550 (920 - 2720)

Data are means ± standard deviation (SD), median (25^th^ to 75^th^ percentile) or absolute counts; ^*^At ICU admission; ^#^average of 373 patient days. GI, gastrointestinal; BMI, body mass index; SOFA, Sequential Organ Failure Assessment; APACHE II, Acute Physiology and Chronic Evaluation II; SAPS II, Simplified Acute Physiology Score II; ICU, intensive care unit.

**Figure 1:**
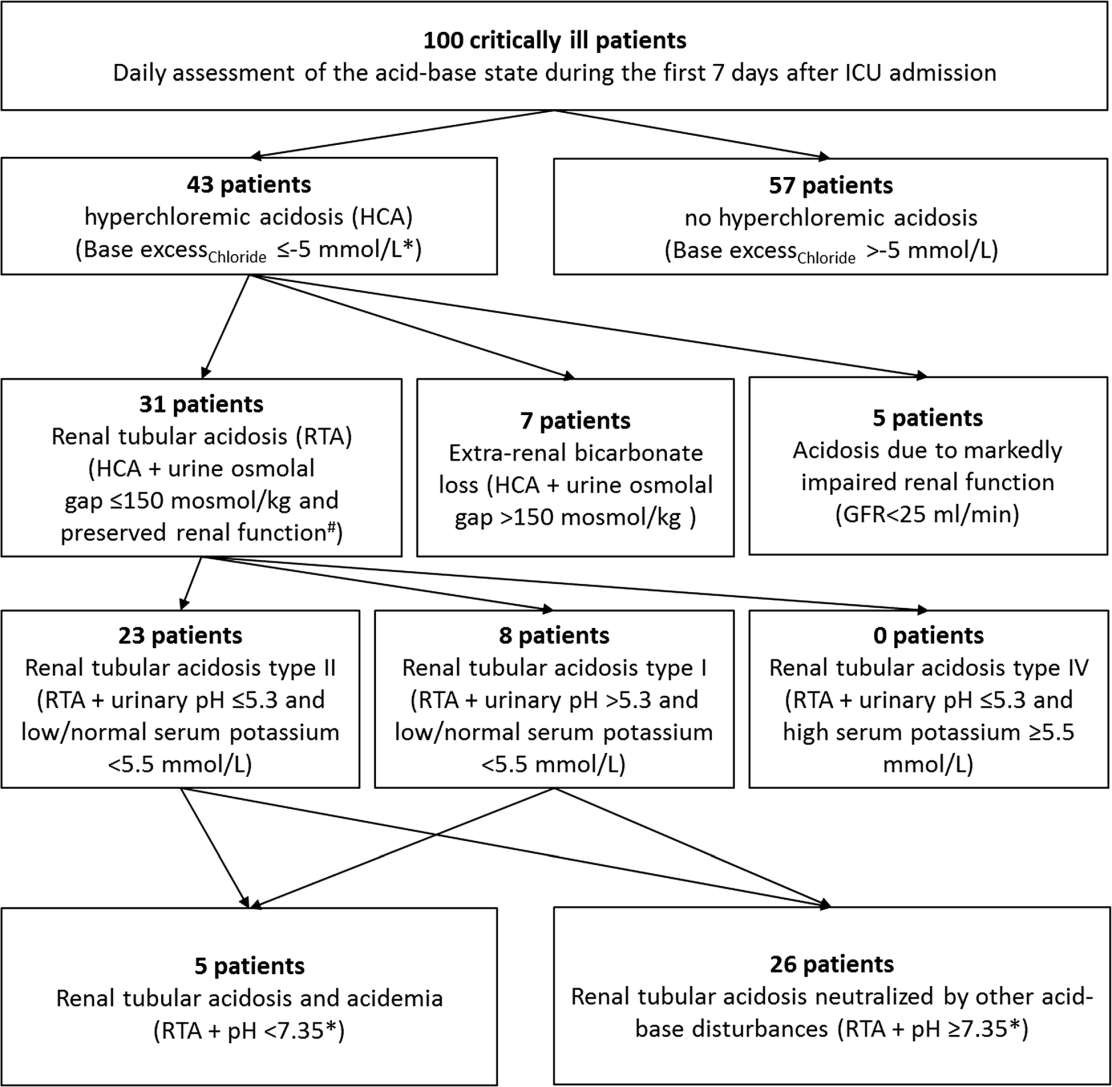
**Daily assessment of the acid-base state during the first 7 days after ICU admission.** Forty-three of the patients presented with hyperchloremic acidosis on one or more days. In 31 patients hyperchloremic acidosis (HCA) was associated with renal-tubular acidosis (RTA) characterized by a urine osmolal gap (UOG) ≤150 mosmol/kg and a preserved renal function. The majority (23 of 31) of patients with RTA presented with RTA type II, while 8 of 31 patients showed characteristics of RTA type I. In 26 of the 31 patients with RTA, metabolic acidosis was neutralized mainly by simultaneously decreased plasma albumin leading to a neutral arterial pH. ^*^On one or more days during the first week after admission; ^#^glomerular filtration rate (GFR) ≥25 ml/min.

**Figure 2:**
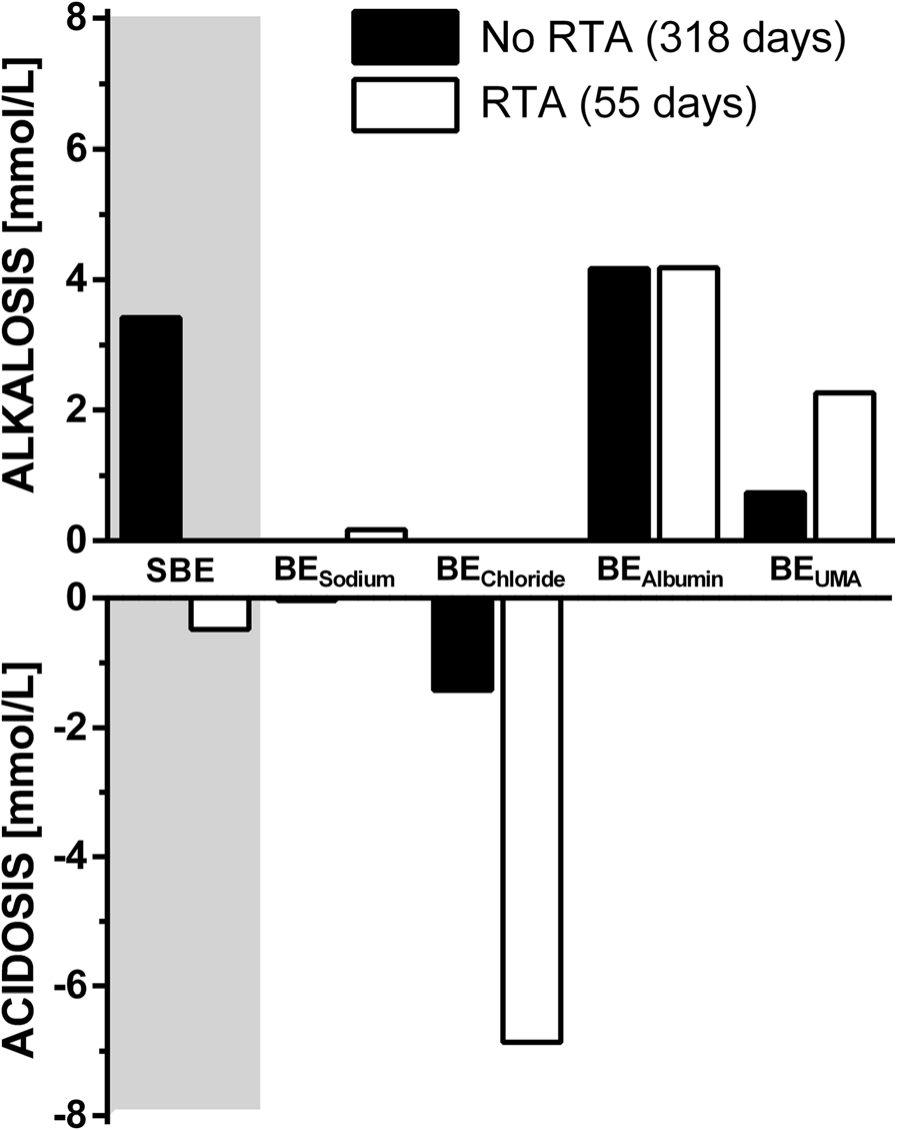
**Standard base excess (SBE) and base excess subsets on days with and without renal tubular acidosis.** Forty-three percent of the patients (86 of 373 patient days) presented with hyperchloremic acidosis on one or more days represented as pronounced negative BE_Chloride_. However, this was frequently neutralized mainly by simultaneously decreased plasma albumin levels resulting in positive BE_Albumin_ and partly by positive BE_UMA_ leading to a neutral arterial pH. In 26 of these 43 patients (55 of 373 patient days) hyperchloremic acidosis was associated with RTA characterized by a UOG of less than 150 mosmol/kg in combination with a preserved renal function. BE_Albumin_, base excess attributable to changes of plasma albumin; BE_Chloride_, base excess attributable to changes of plasma chloride; BE_Sodium_, base excess attributable to changes of free water; BE_UMA_, base excess attributable to unmeasured anions; RTA, renal tubular acidosis; UOG, urine osmolal gap.

During the first week of ICU stay 43 of the patients presented with hyperchloremic acidosis on one or more days represented as pronounced negative BE_Chloride_ (Figure 1). In 31 patients hyperchloremic acidosis was associated with RTA characterized by a UOG of less than 150 mosmol/kg and a preserved renal function. The majority (23 of 31) of patients with RTA presented with RTA type II, while 8 of 31 patients showed characteristics of RTA type I. However, in 26 of the 31 patients with RTA, metabolic acidosis was neutralized mainly by simultaneously decreased plasma albumin leading to a neutral arterial pH (Figures 1 and 2). Moreover, these 26 patients were more ventilated in comparison to the five patients with RTA and acidemia (pCO_2_ 38 ± 4 vs. 46 ± 5 mmHg; *P* <0.001) also contributing to a neutral arterial pH.

Bicarbonate loss was more pronounced in RTA type II in comparison to RTA type I: RTA type II vs. RTA type I SBC 23.9 (22.6 to 25.4) vs. 26 (23.9 to 27) mmol/L; *P* = 0.004.

Of the 31 patients with RTA 18 presented with transient RTA and 13 with persistent RTA. The variable transient vs. persistent RTA was not significantly associated with the clinical outcomes ‘length of ICU stay’, ‘length of hospital stay’, ‘ICU survival’ or ‘hospital survival’.

There were no statistical differences between patients with and without RTA in terms of age, gender, BMI, severity of disease, ICU or hospital outcome, and length of ICU or hospital stay (Table 2).

**Table 2:** **Comparison of demographic and outcome parameters between patients with renal tubular acidosis on one or more days compared to patients without renal tubular acidosis**

	**No RTA (n = 69)**	**RTA (n = 31)**	***P*** **value**
Age (years)	63 ± 16	60 ± 17	0.862
Gender (female/male)	(28/41)	(12/19)	1.000
BMI (kg/m^2^)	26 ± 5	27 ± 4	0.409
SOFA score on admission	8 ± 4	9 ± 4	0.696
APACHE II score on admission	20 ± 9	19 ± 8	0.224
SAPS II score on admission	51 ± 20	52 ± 20	0.837
Length of ICU stay (days)	5 (3–11)	8 (5–11)	0.157
Length of hospital stay (days)	13 (6–22)	16 (8–33)	0.371
ICU mortality (non-survivors [%])	16%	10%	0.404
Hospital mortality (non-survivors [%])	30%	19%	0.332
Deceased within 7 day observation period	10%	6%	0.717

Data are means ± standard deviation (SD), median (25^th^ to 75^th^ percentile) or absolute counts. RTA, renal tubular acidosis; BMI, body mass index; SOFA, Sequential Organ Failure Assessment; APACHE II, Acute Physiology and Chronic Evaluation II; SAPS II, Simplified Acute Physiology Score II; ICU, intensive care unit.

Similarly, in the multivariate analysis with RTA as outcome and diabetes, hypertension, chronic kidney disease, congestive heart failure, liver cirrhosis, neuroleptic drugs, muscle relaxants, combined antibiotic schemes, plasmapheresis, and sedoanalgesia, as well as age, BMI, gender, and severity of illness (SAPS II) as co-factors no variables were predictive for the presence of RTA.

## Discussion

Hyperchloremic metabolic acidosis is a frequent entity in critically ill patients [13]. Therefore, we aimed to identify the underlying acid-base imbalances with a particular focus on RTA. As the occurrence of more than one acid-base disturbance at the same time is common in critically ill patients [11] our analysis was based on calculating the base excess subsets according to the physical-chemical approach by Gilfix *et al.* [12].

Forty-three percent of the patients presented at least on one day with hyperchloremic acidosis during the first week of ICU stay [13]. This is more frequent compared to Gunnerson *et al.* who found hyperchloremic acidosis in only 26% of critically ill patients. However, they did not determine base excess subsets. Therefore, they could not detect hyperchloremic acidosis in the state of normal arterial pH values, which therefore was probably underrepresented [14].

Hyperchloremic metabolic acidosis was frequently neutralized by the simultaneous occurrence of metabolic alkalosis leading to a neutral arterial pH in our patients. The neutralizing effect is mainly attributed to decreased plasma albumin levels resulting in a positive base excess attributable to changes of plasma albumin (BE_Albumin_). The ubiquitous occurrence of hypoalbuminemia in critically ill patients is not seen as counterregulation but is based on the albumin loss due to third space losses, plasma dilution or reduced hepatic production (negative acute phase response) [15-17].

The finding of positive BE_Albumin_ in a large number of the included patients is compatible with the study of Funk *et al.* [11]*.* The second neutralizing effect was based on increased base excess attributable to unmeasured anions (BE_UMA_). The increased BE_UMA_ as seen in our model of acid-base analysis is in theory either caused by an increase in unmeasured cations or a decrease in unmeasured anions. However, our finding of a mildly elevated BE_UMA_ in the ‘No RTA’ group is compatible with the positive BE_UMA_ found in healthy subjects [18]. Therefore, we assume this deviation is caused by a model artifact and the true normal value of BE_UMA_ is +1 mmol/L [11]. However, in the ‘RTA’ group BE_UMA_ was significantly higher in comparison to the ‘No RTA’ group, which can only be explained by the presence of unmeasured cations in patients with RTA. This phenomenon has also been seen in the literature and may be due to the accumulation of guanidines, lithium intoxications, or paraproteinemias with positively charged gammaglobulins [19]. Similarly to Mallet *et al*., the type and origin of unmeasured cations in the RTA group remain open.

To determine the prevalence of RTA we first selected patients with hyperchloremic acidosis (43 of 100 patients; Figure 1). Thirty-one of this subgroup presented with a UOG of less than 150 mosmol/kg in combination with preserved renal function [5], which is characteristic for RTA (Figure 1). Thus, our data show for the first time that RTA was highly prevalent in critically ill patients with hyperchloremic acidosis, which is suggestive that RTA contributes to the pathophysiology of hyperchloremic acidosis in these patients.

Acidemia (arterial pH <7.35) is associated with unfavorable outcome in critically ill patients, which may be pronounced by RTA [14,20]. However, hyperchloremic acidemia is suggested to be less detrimental than lactic or ketoacidemia [21]. Moreover, although there is strong evidence of the association between acidosis and poor outcome in critically ill patients, there is little evidence of causation. Mild (hyperchloremic) acidosis may even be a physiological response as oxygen delivery is enhanced via the Bohr effect [22].

In the present trial, RTA in combination with hyperchloremic acidosis was not associated with the outcome parameters lengths of ICU or hospital stay and ICU or hospital mortality.

Therefore, we hypothesize that RTA in combination with hyperchloremic acidosis in the present study is not a harmful condition, but may be a physiological response in the presence of several metabolic acid-base disorders at the same time (Figure 2).

Hyperchloremic acidosis is attributed to several causes. On the one hand, extrarenal bicarbonate loss may be based on diarrhea or via surgical drains. This is defined as a UOG of more than 150 mosmol/kg and was observed in 16% of the patients with hyperchloremic acidosis (7 of 43) in the present study (Figure 1). Markedly decreased renal function (glomerular filtration rate <25 ml/min [5]) as a potential contributing factor to metabolic acidosis was seen in 12% (5 of 43) of the patients with hyperchloremic acidosis (Figure 1).

Parenterally administered solutions with unphysiologically high chloride content may contribute to hyperchloremic acidosis. This was observed in patients after rapid isotonic saline infusion while undergoing a surgical procedure [23]. Although ‘isotonic’ saline was not used in this study for volume resuscitation, patients may have received it prior to ICU admission. Furthermore, antibiotics and parenteral nutrition contain certain amounts of chloride. On average the amount of sodium chloride applied via medication and parenteral nutrition was rather low and not different between patients with and without RTA (Table 3).

**Table 3:** **Comparison of biochemical parameters on days with and without renal tubular acidosis**

	**No RTA (318 days)**	**RTA (55 days)**	***P*** **value**
Arterial pH	7.41 ± 0.07	7.40 ± 0.19	0.421
pCO_2_	45.3 ± 13.1	39.2 ± 4.9	<0.001
Standard bicarbonate (mmol/L)	27 ± 5	24 ± 2	<0.001
Standard base excess (mmol/L)	3.4 ± 5.1	−0.48 ± 3.51	<0.001
BE_Sodium_ (mmol/L)	0.0 ± 1.4	0.2 ± 1.2	0.317
BE_Chloride_ (mmol/L)	−1.4 ± 4.1	−6.9 ± 1.5	<0.001
BE_Albumin_ (mmol/L)	4.2 ± 1.2	4.2 ± 1.0	0.969
BE_UMA_ (mmol/L)	0.7 ± 2.9	2.3 ± 2.0	<0.001
Serum sodium (mmol/L)	140 ± 5	141 ± 4	0.317
Serum potassium (mmol/L)	4.1 ± 0.5	4.1 ± 0.4	0.894
Serum chloride (mmol/L)	106 ± 5	112 ± 3	<0.001
Serum phosphate (mmol/L)	0.97 ± 0.36	0.89 ± 0.28	0.121
Serum lactate (mmol/L)	1.4 ± 0.9	1.2 ± 0.6	0.319
Urine chloride (mmol/L)	80 ± 42	83 ± 45	0.163
Creatinine clearance (mL/min)	63 (26-106)	83 (46-125)	0.786
NaCl 0.9% infusion per day (mL)	250 (10-550)	300 (114-857)	0.268

Data are means ± standard deviation (SD), median (25^th^ to 75^th^ percentile) or absolute counts. RTA, renal tubular acidosis; BE_Sodium_, base excess attributable to changes of free water; BE_Chloride_, base excess attributable to changes of plasma chloride; BE_Albumin_, base excess attributable to changes of plasma albumin; BE_UMA_, base excess attributable to unmeasured anions.

However, despite the significantly increased blood chloride levels in patient samples with RTA, urine chloride levels were not elevated (Table 3). This suggests an altered renal chloride handling as seen in RTA rather than external chloride administration.

Hyperchloremia *per se* is known to cause gastrointestinal symptoms, reduced renal blood flow and glomerular filtration rate, as well as coagulation disorders [22]. Whereas coagulation disorders and gastrointestinal symptoms were not studied in the present trial, hyperchloremic acidosis *per se* - independent of the resulting arterial pH - was not associated with decreased glomerular filtration rate or with increased length of stay or mortality (data not shown).

Ammonium chloride-loading/frusemide tests and the bicarbonate-loading test are the golden standards to distinguish between proximal and distal RTA in non-critically ill patients [3]. However, due to potential gastrointestinal, renal, and respiratory side effects of these drugs we decided to use a non-invasive approach based on urinary pH and urinary potassium [9].

A potential weakness of our study is the use of the UOG as a surrogate for urinary ammonium concentrations. However, the direct measurement of urinary ammonium was not routinely available in our hospital [24].

Another weakness of the present trial is that it was only a single-center observation with all its known limitations [25].

Moreover, the findings derived from our study are only associative and are, therefore, naturally only hypothesis generating.

To compensate for shortcomings in the present trial, we suggest that future trials should be conducted in a multicenter setting, should be based on direct measurement of urinary ammonia, should also focus on unmeasured cations, and should prospectively collect data on multiorgan dysfunction, effectiveness of therapies administered, and neurological/cardiac performance.

Hypoalbuminemia is ubiquitous in critically ill patients represented by positive BE_Albumin_. Thus, RTA - in the presence of normal arterial pH - may be a physiological response and the result of the counterregulation of metabolic alkalosis due to low albumin levels.

## Conclusions

RTA is highly prevalent in critically ill patients with hyperchloremic acidosis, whereas it is often neutralized by the simultaneous occurrence of other acid-base disturbances.

## Key messages

- Hyperchloremic acidosis and renal tubular acidosis are highly prevalent in critically ill patients
- The simultaneous occurrence of multiple acid-base disturbances at the same time in the same patient is common in the critically ill

## Abbreviations

APACHE II: Acute Physiology and Chronic Evaluation II
BE: base excess
BE_Albumin_: base excess attributable to changes of plasma albumin
BE_Chloride_: base excess attributable to changes of plasma chloride
BE_Sodium_: base excess attributable to changes of free water
BE_UMA_: base excess attributable to unmeasured anions
BMI: body mass index
GFR: glomerular filtration rate
HCA: hyperchloremic acidosis
ICU: intensive care unit
RTA: renal tubular acidosis
SAG: serum anion gap
SAPS II: Simplified Acute Physiology Score II
SBE: standard base excess
SOFA: Sequential Organ Failure Assessment
UOG: urine osmolal gap
